# Effects of ectomycorrhizal fungi (*Suillus variegatus*) on the growth, hydraulic function, and non-structural carbohydrates of *Pinus tabulaeformis* under drought stress

**DOI:** 10.1186/s12870-021-02945-3

**Published:** 2021-04-10

**Authors:** Jiaxing Wang, Haoqiang Zhang, Jing Gao, Yu Zhang, Yaqin Liu, Ming Tang

**Affiliations:** 1 College of Forestry, Northwest A&F University, Yangling, 712100 People’s Republic of China; 2 State Key Laboratory for Conservation and Utilization of Subtropical Agro-Bioresources, Lingnan Guangdong Laboratory of Modern Agriculture, Guangdong Key Laboratory for Innovative Development and Utilization of Forest Plant Germplasm, College of Forestry and Landscape Architecture, South China Agricultural University, Guangzhou, 510642 People’s Republic of China

**Keywords:** Ectomycorrhizal fungi, *Pinus tabulaeformis*, Drought stress, Non-structural carbohydrate, Forest restoration

## Abstract

**Background:**

A better understanding of non-structural carbohydrate (NSC) dynamics in trees under drought stress is critical to elucidate the mechanisms underlying forest decline and tree mortality from extended periods of drought. This study aimed to assess the contribution of ectomycorrhizal (ECM) fungus (*Suillus variegatus*) to hydraulic function and NSC in roots, stems, and leaves of *Pinus tabulaeformis* subjected to different water deficit intensity. We performed a continuous controlled drought pot experiment from July 10 to September 10, 2019 using *P. tabulaeformis* seedlings under 80, 40, and 20% of the field moisture capacity that represented the absence of non-drought, moderate drought, and severe drought stress, respectively.

**Results:**

Results indicated that *S. variegatus* decreased the mortality rate and increased height, root biomass, and leaf biomass of *P. tabulaeformis* seedlings under moderate and severe drought stress. Meanwhile, the photosynthetic rates, stomatal conductance, and transpiration rates of *P. tabulaeformis* were significantly increased after *S. variegatus* inoculation. Moreover, the inoculation of *S. variegatus* also significantly increased the NSC concentrations of all seedling tissues, enhanced the soluble sugars content, and increased the ratios of soluble sugars to starch on all tissues under severe drought. Overall, the inoculation of *S. variegatus* has great potential for improving the hydraulic function, increasing the NSC storage, and improving the growth of *P. tabulaeformis* under severe drought.

**Conclusions:**

Therefore, the *S. variegatus* can be used as a potential application strain for ecological restoration on arid regions of the Loess Plateau, especially in the *P. tabulaeformis* woodlands.

**Supplementary Information:**

The online version contains supplementary material available at 10.1186/s12870-021-02945-3.

## Background

Water deficit is one of the most important environmental stresses affecting plants productivity and reducing grain yield around the world [1–4]. The drought-induced tree mortality throughout the world have been documented over the last several years [5, 6], and an even greater drought-induced loss of net ecosystem productivity is the nonlethal reductions in growth [7–9]. The Loess Plateau is the most severe drought and soil erosion area in the world [10]. In the past 30 years, *Pinus tabulaeformis*, which can form a symbiotic relationship with ectomycorrhizal (ECM) fungi, has been planted on a large area on the Loess Plateau to maintain soil and water and to improve the ecological environment [11–13]. However, a large number of *P. tabulaeformis* died due to the drought on the Loess Plateau. Since plants cannot relocate, their survival largely depends on the tolerance and rapid responses to counter the stress effects [14]. Consequently, it is urgent to conduct study to improve the survival rate of *P. tabulaeformis* and increase its drought resistance in this area.

To alleviate the negative effects of drought on plants, the influence of microorganisms on host plants has received extensive attention. Mycorrhizal fungi improve the ability of their hosts to resist, tolerate, and recover from drought [15, 16]. The extrametrical fungal hyphae can deeply extend into the rhizosphere of the soil leading to the absorption of large amounts of various nutrients, increasing the availability and uptake of micro-nutrients, and alleviating the stress effects on plant [17]. Previous studies have shown that the water transport via extraradical mycelium of ECM fungi to host plant can be sufficient to make a difference between the survival and death of a tree seedling [18]. Lehto and Zwiazek [16] concluded that the influences of ECM fungi on host-plant–water relations were manifested in increased stomatal conductance to water vapor, altered hydraulic conductance of mycorrhizal roots, and facilitated osmotic adjustments. However, the physiological responses of plants to inoculation with ECM fungi were highly variable and dependent upon the species and even genotype of fungus [7].

Previous studies have shown that *P. tabulaeformis* could form a mycorrhizal symbiosis relationship with *Suillus*, *Tomentella*, *Tuber*, *Handkea* etc. [19–21]. Meanwhile, we investigated the fungal resources of the *P. tabulaeformis* forests on the Loess Plateau and found that *Suillus variegatus* (Swartz ex Fr.) O. Kuntze and *P. tabulaeformis* had a close symbiotic relationship. *S. variegatus* is an epigeous ECM fungal species that is widespread and common in pine forests in Europe and in parts of Asia [22, 23]. And *S. variegatus* can form an ECM symbiosis system with *Pinus sylvestris* L. seedlings [24], promoting the growth of roots and shoots of seedlings [25]. Inoculation with *S. variegatus* also increased the chitin content of *P. sylvestris* seedlings and the contents of Mg and K in the rhizosphere soil. However, few studies have clarified the specific effects of *S. variegatus* on the growth of *P. tabulaeformis*.

As the photosynthesis is one of the most important physiological processes for plants to obtain carbon [26]. When drought stress causes a decrease in photosynthesis, trees are vulnerable to carbon starvation [27]. Prolonged drought stress will significantly reduce the storage of non-structural carbohydrates (NSC) in the trees, and eventually lead to death [8, 28]. The NSC, which play different roles in plant energy metabolism, transportation and osmotic adjustment, is mainly starch and soluble sugars [29]. Starch is the most prevalent and abundant storage carbohydrate in woody tissues [30, 31]. When plants suffered from severe drought stress that causes carbohydrate deficiencies, starch would be consumed in large quantities and be preferentially transported to growing parts. Soluble sugars also play an important role in osmotic regulation and are considered to be important physiological indicators related to drought tolerance [2, 32]. Therefore, the NSC could make a buffer between carbohydrate supply and demand, and allow trees to resist drought [33]. Mycorrhizal hyphal are dynamic and play a crucial role in forest ecosystem functioning and carbon dioxide fluxes [34, 35]. However, mycorrhizal hyphal usually come at the cost of increased carbohydrate costs [18]. In ECM systems, fungi have been reported to receive up to 19 times more carbohydrates, which results in a strong belowground carbon sink [33], from the host tree compared with the normal exudation of root systems [36]. However, the effect of ECM on plant’s NSC content under different levels of drought intensity has not been fully understood.

Though there are many studies on the effect of different ECM fungi on different plant species, no information is available on the effect of *S. variegatus* inoculation on the growth and NSC content of *P. tabulaeformis* under drought conditions. The hypothesis of the present study was that the *S. variegatus* could form a close symbiotic relationship with *P. tabulaeformis* seedlings, promote the growth, reduce mortality, and participate in regulating the content of NSC in tissues under drought conditions. Therefore, to test this hypothesis, we conducted a 6-month drought-simulating indoor pot experiment. The biomass and mortality of *P. tabulaeformis* were used to evaluate plant growth. The starch and soluble sugar content, and their ratio in various tissues of *P. tabulaeformis* were used to study the dynamic changes of NSC. In addition, the gas exchange parameters were used to interpret the mechanisms involved in NSC variations caused by *S. variegatus* under drought stress.

## Results

### The *S. variegatus* colonization of *P. tabulaeformis*

At harvest, non-inoculated *P. tabulaeformis* seedlings did not been colonized and all inoculated seedlings been colonized by *S. variegatus* in their root systems. The seedlings in the severe drought soil had the lowest and in the moderate drought soil had the highest ECM fungus colonization, at 38 and 60%, respectively. (Table 1).

**Table 1:** The soil water content and ECM colonization of *Pinus tabulaeformis* seedlings under different drought intensity treatments. (mean ± standard error)

Parameter	T1	T2	T3	Significance
Soil water content (%)	80	40	20	
ECM colonization (%)	52 ± 6 b	60 ± 3 a	38 ± 6 c	**

Note: T1 = non-drought stress, T2 = moderate drought stress, T3 = severe drought stress. Data expressed as mean ± standard error (*n* = 6). Different lowercase letters indicate significant differences between the means by Tukey (HSD) test (*P* < 0.05); “*” indicates that the interaction is significant (*P*<0.05); “**” indicates that the interaction is extremely significant (*P*<0.01); “ns” indicates no interaction (*P* ≥ 0.05)

### The mortality rate and growth of *P. tabulaeformis*

**Fig. 1:**
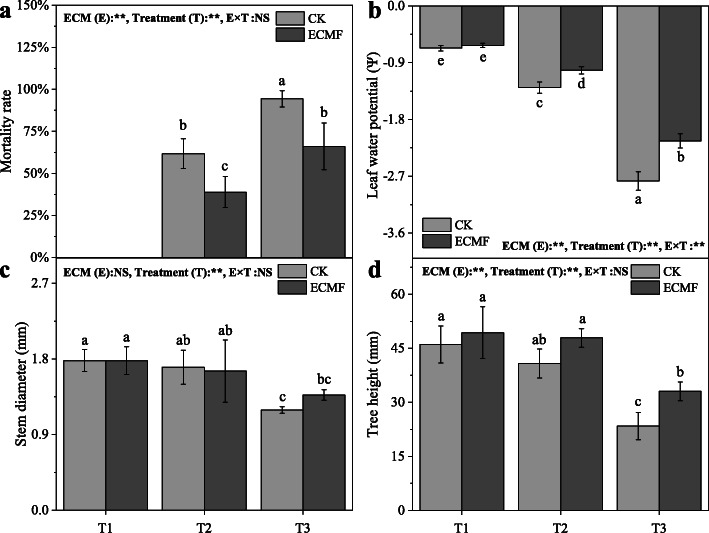
The mortality rate (**a**), leaf water potential (**b**), stem diameter (**c**) and tree height (**d**) of *Pinus tabulaeformis* with ECM fungi inoculation in three drought levels. The data are the means ± standard deviation (*n* = 3). Different lowercase above the columns indicate significant difference between the means by Tukey (HSD) test (*P* < 0.05). CK = No ECM fungi inoculation; ECMF = ECM fungi inoculation; T1 = non-drought stress; T2 = moderate drought stress; T3 = severe drought stress

The mortality rate of *P. tabulaeformis* seedlings increased significantly (*P* < 0.05) as the drought gradient increased (Fig. 1A). In non-drought (T1) soil, all seedlings survived. In the T2, T3 treatment, the inoculation of *S. variegatus* could significantly reduce (*P* < 0.05) the mortality, by 20%, compared with the mortality of non-inoculation.

The leaf water potential of *P. tabulaeformis* seedlings decreased significantly (*P* < 0.05) as the drought intensity increased (Fig. 1B). The inoculation of *S. variegatus* could significantly increase (*P* < 0.05) the leaf water potential among the T2 and T3 drought intensity.

The stem diameter of *P. tabulaeformis* seedlings decreased significantly (*P* < 0.05) in T3 treatment (Fig. 1C). There was no significant difference in the stem diameter of seedlings between non-inoculated and inoculated in all treatments. Like stem diameter, the height of *P. tabulaeformis* seedlings decreased significantly (*P* < 0.05), but the inoculation of *S. variegatus* could significantly increase (*P* < 0.05) the height compared with non-inoculated in T3 treatment (Fig. 1D). With the drought gradient increased, seedlings stem diameter and height showed a significant downward trend (*P* < 0.05).

### The root index of *P. tabulaeformis*

The drought gradient and *S. variegatus* inoculation had a significant effect on the root index of *P. tabulaeformis* seedlings (Table 2). Data showed with the increase of drought intensity, all root indexes decreased except the root average diameter. The inoculation of *S. variegatus* could significantly increase the root length, surface area and root volume under non-drought (T1 treatment) compared with CK group. Under T2 treatment, the root length, surface area and forks of seedlings with *S. variegatus* inoculation were significantly higher than CK group. And the root length and surface area were greatly increased by *S. variegatus* inoculation under severe drought (T3 treatment).

**Table 2:** Effect of ECM fungi on root indicators of *Pinus tabulaeformis* seedlings under different drought intensity treatments. (mean ± standard error)

Drought intensity treatments (T)	ECM fungi (E)	Length (cm)	Surface area (cm^2^)	Average diameter (mm)	Root volume (cm^3^)	Tips	Forks
T1	Non-ECM	338.6 ± 13.91 b	55.95 ± 1.28 b	0.54 ± 0.02 c	0.74 ± 0.06 b	845 ± 284 ab	1398 ± 113 a
	ECM	460.94 ± 59.85 a	76.55 ± 12.07 a	0.53 ± 0.01 c	1.01 ± 0.19 a	1009 ± 197 a	1728 ± 322 a
T2	Non-ECM	194.59 ± 29.15 c	40.76 ± 8.37 c	0.66 ± 0.05 a	0.68 ± 0.18 b	572 ± 25 bcd	835 ± 127 b
	ECM	346.8 ± 43.1 b	56.75 ± 6.9 b	0.53 ± 0.02 c	0.74 ± 0.08 bc	826 ± 126 abc	1500 ± 296 a
T3	Non-ECM	107.95 ± 5.18 d	20.9 ± 1.6 d	0.63 ± 0.01 ab	0.32 ± 0.03 c	299 ± 57 c	487 ± 41 b
	ECM	173.74 ± 26.92 c	31.83 ± 3.92 c	0.59 ± 0.02 bc	0.47 ± 0.04 cd	409 ± 98 cd	708 ± 127 b
Effect (*P* value)	T	**	**	**	**	**	**
	E	**	**	**	*	*	**
	T & E	ns	ns	**	ns	ns	ns

Note: T1 = non-drought stress, T2 = moderate drought stress, T3 = severe drought stress. Data expressed as mean ± standard error (*n* = 6). Different lowercase letters indicate significant differences between the means by Tukey (HSD) test (P < 0.05); “*” indicates that the interaction is significant (*P*<0.05); “**” indicates that the interaction is extremely significant (*P*<0.01); “ns” indicates no interaction (*P* ≥ 0.05)

### The biomass of various tissues of *P. tabulaeformis*

**Fig. 2:**
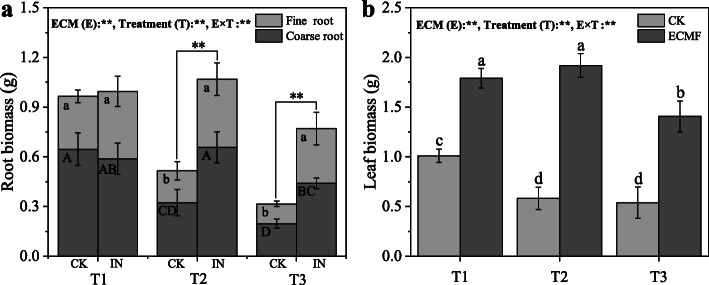
The root (**a**) and leaf biomass (**b**) of *Pinus tabulaeformis* with ECM fungi inoculation in three drought levels. The data are the means ± standard deviation (*n* = 3). Different lowercase above the columns indicate significant difference between the means by Tukey (HSD) test (*P* < 0.05). CK = No ECM fungi inoculation; ECMF = ECM fungi inoculation; T1 = non-drought stress; T2 = moderate drought stress; T3 = severe drought stress

The seedlings in severe drought soil had lower root and leaf biomass compared with the seedlings in the T1 and T2 treatment (Fig. 2). The inoculation of *S. variegatus* could significantly increase the fine root biomass, by 116 and 175%, respectively, compare with CK group in the T2, T3 treatments (Fig. 2A). And it also could significantly increase the coarse roots biomass, by 97 and 120%, respectively, compare with CK group in the T2 and T3 treatment. Therefore, the root biomass was significantly increased (*P* < 0.01) after *S. variegatus* inoculation, by 106 and 140%, respectively, compare with non-inoculation in the T2 and T3 treatment. Like fine root biomass, the leaf biomass also greatly increased, by 63, 210 and 160%, respectively, after *S. variegatus* inoculation in three treatments (Fig. 2B). However, drought and *S. variegatus* inoculation had no significant effect on the biomass of the stem (Table 3).

**Table 3:** Effect of ECM fungi on some indicators of *Pinus tabulaeformis* seedlings under different drought intensity treatments. (mean ± standard error)

Drought intensity treatments (T)	ECM fungi (E)	Stem biomass (mg)	Stem water content (%)	Stomatal conductance (mmol H_2_O m^−2^ s^−1^)	Intercellular CO_2_ concentration (μmol CO_2_ mol^− 1^)
T1	Non-ECM	108 ± 10 a	56 ± 15 b	0.28 ± 0.10 a	362 ± 19 a
	ECM	195 ± 77 a	56 ± 3.6 b	0.29 ± 0.10 a	357 ± 11 a
T2	Non-ECM	110 ± 58 a	61 ± 10 a	0.04 ± 0.01 b	315 ± 66 a
	ECM	168 ± 91 a	63 ± 4.3 a	0.12 ± 0.05 b	325 ± 26 a
T3	Non-ECM	119 ± 10 a	32 ± 6.2 c	0.02 ± 0.01 c	308 ± 67 a
	ECM	191 ± 20 a	37 ± 7.3 c	0.04 ± 0.01 b	329 ± 48 a
Effect (*P* value)	T	ns	**	**	*
	E	ns	ns	*	ns
	T & E	ns	ns	ns	ns

Note: T1 = non-drought stress, T2 = moderate drought stress, T3 = severe drought stress. Data expressed as mean ± standard error (*n* = 6). Different lowercase letters indicate significant differences between the means by Tukey (HSD) test (P < 0.05); “*” indicates that the interaction is significant (*P*<0.05); “**” indicates that the interaction is extremely significant (*P*<0.01); “ns” indicates no interaction (*P* ≥ 0.05)

### The water content of root and leaf of *P. tabulaeformis*

**Fig. 3:**
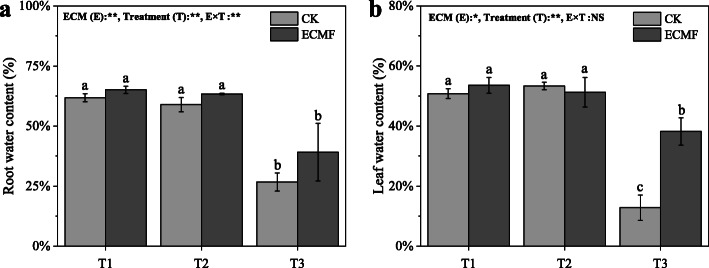
The root (**a**) and leaf water content (**b**) of *Pinus tabulaeformis* with ECM fungi inoculation in three drought levels. The data are the means ± standard deviation (*n* = 3). Different lowercase above the columns indicate significant difference between the means by Tukey (HSD) test (*P* < 0.05). CK = No ECM fungi inoculation; ECMF = ECM fungi inoculation; T1 = non-drought stress; T2 = moderate drought stress; T3 = severe drought stress

The seedlings in severe drought soil had lower root and leaf water content compared with the seedlings in the T1 and T2 treatment (Fig. 3). The inoculation of *S. variegatus* could significantly increase the leaf water content, by 216%, compare with CK group in T3 treatment. However, the highest and lowest stem water content was in T2 and T3 treatment, respectively (Table 3). There was no significant effect on the water content of the stem after *S. variegatus* inoculation.

### The leaf gas exchange parameters of *P. tabulaeformis*

**Fig. 4:**
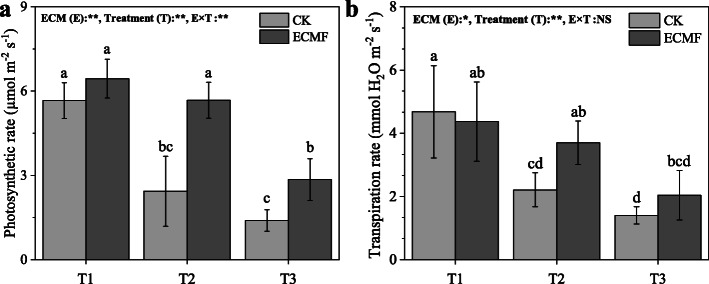
The photosynthetic (**a**) and transpiration rate (**b**) of *Pinus tabulaeformis* with ECM fungi inoculation in three drought levels. The data are the means ± standard deviation (*n* = 3). Different lowercase above the columns indicate significant difference between the means by Tukey (HSD) test (*P* < 0.05). CK = No ECM fungi inoculation; ECMF = ECM fungi inoculation; T1 = non-drought stress; T2 = moderate drought stress; T3 = severe drought stress

The seedlings in severe drought soil had lower photosynthetic rate and transpiration rate compared with the seedlings in the T1 and T2 treatment (Fig. 4). The inoculation of *S. variegatus* could significantly increase photosynthetic rate, by 133 and 100%, respectively, compare with CK group in T2 and T3 treatment (Fig. 4A). Meanwhile, the inoculation of *S. variegatus* also increased transpiration rate, by 67%, compare with CK group in T2 treatment (Fig. 4B).

Stomatal conductance also greatly increased with *S. variegatus* inoculation in T3 treatment (Table 3). However, no significant effect of ECM inoculation on intercellular CO_2_ concentration was found under all treatments.

### The non-structural carbohydrate of *P. tabulaeformis*

**Fig. 5:**
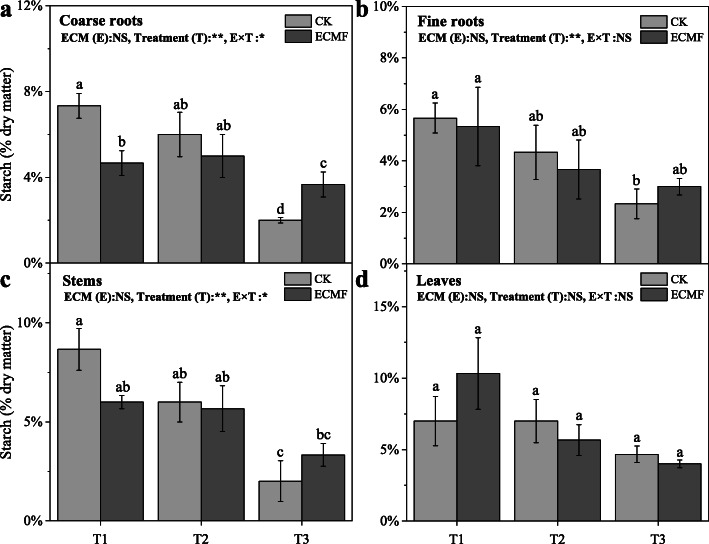
The starch content of different tissues (coarse roots (**a**); fine roots (**b**); stems (**c**); leaves (**d**)) of *Pinus tabulaeformis* with ECM fungi inoculation in three drought levels. The data are the means ± standard deviation (*n* = 3). Different lowercase above the columns indicate significant difference between the means by Tukey (HSD) test (*P* < 0.05). CK = No ECM fungi inoculation; ECMF = ECM fungi inoculation; T1 = non-drought stress; T2 = moderate drought stress; T3 = severe drought stress

As the drought intensity increased, the starch content of seedling all tissues decreased significantly (Fig. 5). The seedlings tissues in severe drought soil had lower starch content compared with the seedlings in the T1 and T2 treatment. The inoculation of *S. variegatus* could significantly increase the coarse roots starch content, by 80%, compare with CK group in T3 treatment (Fig. 5A). However, no significant effect of *S. variegatus* inoculation on other tissues was found under all treatments.

**Fig. 6:**
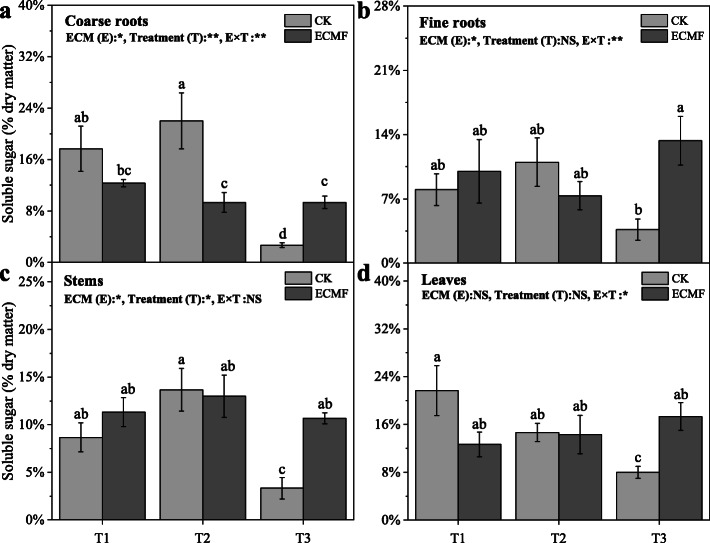
The soluble sugar content of different tissues (coarse roots (**a**); fine roots (**b**); stems (**c**); leaves (**d**)) of *Pinus tabulaeformis* with ECM fungi inoculation in three drought levels. The data are the means ± standard deviation (*n* = 3). Different lowercase above the columns indicate significant difference between the means by Tukey (HSD) test (*P* < 0.05). CK = No ECM fungi inoculation; ECMF = ECM fungi inoculation; T1 = non-drought stress; T2 = moderate drought stress; T3 = severe drought stress

**Fig. 7:**
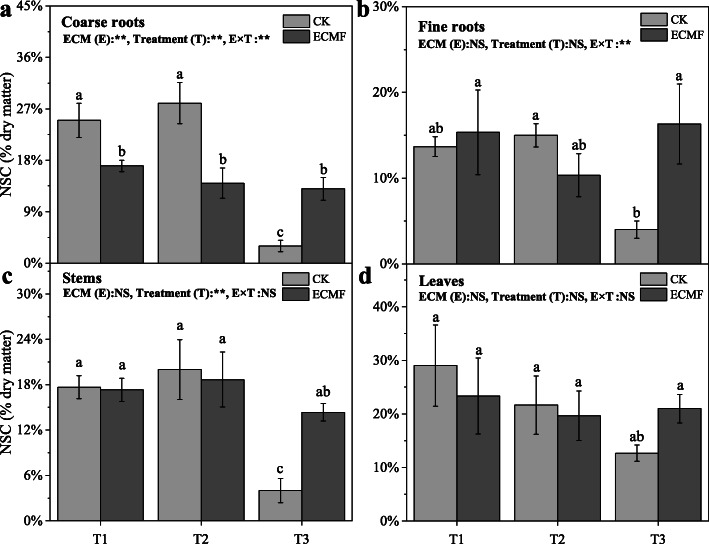
The non-structural carbohydrate (NSC) content of different tissues (coarse roots (**a**); fine roots (**b**); stems (**c**); leaves (**d**)) of *Pinus tabulaeformis* with ECM fungi inoculation in three drought levels. The data are the means ± standard deviation (*n* = 3). Different lowercase above the columns indicate significant difference between the means by Tukey (HSD) test (*P* < 0.05). CK = No ECM fungi inoculation; ECMF = ECM fungi inoculation; T1 = non-drought stress; T2 = moderate drought stress; T3 = severe drought stress

The soluble sugar content of seedling all tissues decreased significantly in non-inoculation group, remained stable in inoculation group under three treatments (Fig. 6). The inoculation of *S. variegatus* could significantly increase the soluble sugar content of coarse roots, fine roots, stems and leaves, by 800, 550, 614 and 113%, respectively, compare with non-inoculation group in T3 treatment (Fig. 6). This change trend was similar to the change in NSC content, the NSC content of seedling all tissues decreased significantly in non-inoculation group, remained stable in inoculation group under three treatments. The inoculation of *S. variegatus* could significantly increase the NSC content of coarse roots, fine roots, stems and leaves, by 333, 300, 250 and 62%, respectively, compare with CK group in T3 treatment (Fig. 7).

**Fig. 8:**
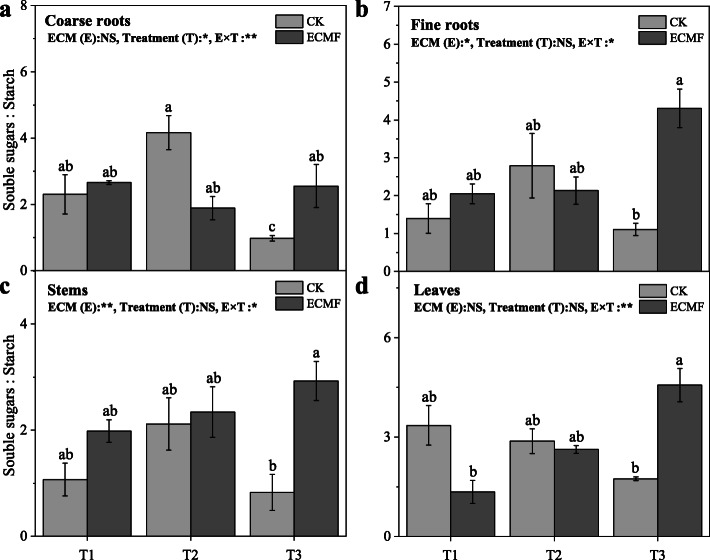
The ratios of soluble sugars to starch of different tissues (coarse roots (**a**); fine roots (**b**); stems (**c**); leaves (**d**)) of *Pinus tabulaeformis* with ECM fungi inoculation in three drought levels. The data are the means ± standard deviation (*n* = 3). Different lowercase above the columns indicate significant difference between the means by Tukey (HSD) test (*P* < 0.05). CK = No ECM fungi inoculation; ECMF = ECM fungi inoculation.; T1 = non-drought stress; T2 = moderate drought stress; T3 = severe drought stress

The non-inoculation seedling tissues in severe drought soil had lower the ratios of soluble sugars to starch compared with the seedlings in the T1 and T2 treatment (Fig. 8). The inoculation of *S. variegatus* could significantly increase the ratios of soluble sugars to starch of coarse roots, fine roots, stems, and leaves compare with non-inoculation group in T3 treatment. Despite drought intensity, the ratios of soluble sugars to starch in coarse roots tended to stable throughout the experiment, while that in all other tissues tended to increase (Fig. 8).

## Discussion

Severe drought causes significant declines in the productivity and survival of plants [37–39]. In our study, the mortality of *P. tabulaeformis* seedlings was more than 95% under severe drought condition, and was decreased by the *S. variegatus* inoculation. Two mechanisms have been proposed to explain drought-induced tree mortality: hydraulic failure and carbon starvation [40]. Therefore, the present experimental results show that the inoculation of *S. variegatus* may alleviate hydraulic failure and carbon starvation caused by drought. The present study also showed that *S. variegatus* inoculation increased plant height, stem diameter, root (both coarse and fine roots) biomass, and leaf biomass. This might be due to the increased absorption area of the plant roots in soil by establishment of the ECM symbiosis [41, 42].

The strategies of plants resist drought rely on hydraulic mechanisms: reducing water loss, and increasing water uptake [38, 43]. Under drought conditions, plants would significantly reduce leaf water potential and gas exchange [44, 45]. As an isohydric species, *P. tabulaeformis* can reduce stomatal conductance and maintain a relatively constant leaf water potential during low water availability periods [27, 46, 47]. Previous studies had shown that the leaf water potential values between − 1.4 and − 1.5 MPa can represent the carbon safety margin of isotonic tree species [48]. In our study, the average of leaf water potential of *P. tabulaeformis* in the control group was − 1.29 (± 0.09) MPa and − 2.78 (± 0.15) MPa under moderate and severe drought conditions, respectively. This means that *P. tabulaeformis* was already in a state of carbon starvation under moderate drought conditions, while under severe drought conditions, it would easily die from severe carbon starvation. These were consistent with our mortality test results. However, the leaf water potential of inoculation group was significantly higher than that of the control, maintaining at − 1.02 (± 0.06) MPa and − 2.14 (± 0.11) MPa under moderate and severe drought conditions, respectively. Therefore, we found that the inoculation of *S. variegatus* could increase the leaf water potential to maintain a safe range (especially under moderate drought conditions), thereby alleviating the damage caused by drought and reducing the mortality of trees. At the same time, *S. variegatus* inoculation in moderate and severe droughts could significantly increase the stomatal conductance, transpiration rate, and photosynthetic rate of plants. The influence suggested the participation of *S. variegatus* in the hydraulic adjustments of *P. tabulaeformis* under drought. The high stomatal conductance at low soil moisture content might be due to the extraction of soil moisture by mycorrhizal system or the expansion of exploration area by extra-root hyphae [49]. At the same time, the increment of stomatal conductance increases the absorption of CO_2_, and consequently increases the photosynthesis rate of plants [7, 50]. In this way, although the *P. tabulaeformis* is still restricted by the stomatal conductance under drought conditions, it would produce more carbohydrates required for respiration than the control, thereby maintaining basic metabolic and defensive capabilities. Taken together, we suggested that *S. variegatus* inoculation improves the photosynthesis of *P. tabulaeformis* under drought through increased water absorption and transportation.

Trees are vulnerable to carbon starvation induced by drought stress [51]. Previous studies have shown that severe, but not moderate, drought stress would significantly influence NSC concentration [52, 53], which is similar to our results. When *P. tabulaeformis* suffers severe drought, the starch content of other tissues except the leaves will be significantly reduced. As NSC have been identified as key carbon sources under severe drought [54, 55], decreasing starch concentrations of tissues indicate the remobilization of starch reserves in *P. tabulaeformis* seedlings in order to meet C requirements when drought response reduces C assimilation [56]. The insignificant decrease in the content of starch in leaves may be due to it initially declined, and then increased above pre-drought concentrations before mortality [57]. The inoculation of *S. variegatus* could significantly increase the NSC concentrations of all seedling tissues compared with the control group. This phenomenon suggested that *S. variegatus* could improve the NSC storage and alleviate carbon starvation of *P. tabulaeformis* under severe drought. This may be due to the close relationship between plant stomatal conductance, leaf hydraulic regulation and C reserve under drought conditions [58]. In our study, inoculation with *S. variegatus* could directly increase the stomatal conductance and leaf water potential, which means that seedlings could perform more photosynthesis. Although the increase in stomatal conductance may increase the loss of water, from our experimental results, the increase of photosynthesis was beneficial to seedlings under drought. Meanwhile, the inoculation of *S. variegatus* had different effects on the composition of seedling NSC. That maybe due to the NSC reserves can adjust their tissue concentrations in response to changes in the balance of C sources or sinks [59, 60]. On the one hand, the inoculation of *S. variegatus* could increase the starch content, which can be preferentially transported to the growing part of the plant roots to ensure root growth, in crude roots under severe drought conditions. On the other hand, the inoculation of *S. variegatus* could increase all tissues’ soluble sugar content and the ratio of soluble sugar to starch under severe drought conditions, which can reduce the water potential, maintain cell expansion, and increase water absorption [61]. Therefore, we believe that *S. variegatus* inoculation would play an important role in alleviating C starvation caused by drought in *P. tabulaeformis*.

## Conclusion

The inoculation of *S. variegatus* could greatly promote the growth and survival rate of *P. tabulaeformis* by increasing the water absorption and transportation, and improving the NSC storage under severe drought. In general, the *S. variegatus* have the potential to be used as biological modifiers in ecological restoration on arid regions of the Loess Plateau, especially in the *P. tabulaeformis* woodlands.

## Materials and methods

### Plant material and growth substrate

*P. tabulaeformis* trees were grown from seed in nursery trays at the microbiology lab of the Forestry College, Northwest A&F University. The seeds were provided by the Forestry Technology Extension Station of the Forestry Department, Shaanxi Province, China. The seeds were sterilized on the surface with 0.05% KMnO_4_ for 30 min, washed 3 times with sterilized water, and immersed in sterilized water at 45 °C for 1 h. The sterilized seeds are placed in sterile gauze and cultured under sterile dark conditions. The sterilized seeds were germinated in sterile gauze at 25 °C under sterile dark conditions. The germinated seeds were transplanted into seedling trays (50 mL per hole) filled with sterilized vermiculite. The seeds were fertilized every week with 10 mL of 1/2-strength Hoagland’s solution [62]. In March 2019, 180 2-month seedlings with a height to meristem of 30 ± 5 mm and a root collar diameter of 1 ± 0.4 mm were selected and transplanted into plastic pots ( 10 cm in diameter, 10 cm in depth) containing 2 kg of growth substrate. Each pot was planted with 3 seedlings, for a total of 60 pots. The seedlings were kept under optimal growth conditions (ambient light, 24 °C, RH = 70%, ultra-optimal irrigation) for a 4-week acclimation period.

The growth substrate of *P. tabulaeformis* was composed of a mixture of soil, sand, and vermiculite (1: 1: 1, v/v/v). Soil was collected from the top layer of the Northwest A&F University campus nursery in Yangling city, Shaanxi province, China. The main soil nutrient characteristics were as follows: 16.15 g kg^− 1^ organic matter, 30.35 mg kg^− 1^ available nitrogen, 20.40 mg kg^− 1^ available phosphorus and 126.36 mg kg^− 1^ available potassium. Soil was ground, passed through a 2-mm sieve, and mixed with thoroughly washed river sand and vermiculite. The mixture was autoclaved at 0.11 MPa and 121 °C for 2 h, after which it sat for 1 week before use.

### Fungal preparation and inoculation

ECM fungal inoculum (*S. variegatus*) was stored in the microbiology lab of the Forestry College, Northwest A&F University. The strain was originally isolated from the ectomycorrhizae of *P. tabulaeformis* on the Loess Plateau. The fungus was firstly cultivated on potato dextrose agar (PDA) solid medium. After 2 weeks of growth, four blocks of media (1 cm in diameter) were inoculated in every 300 ml potato dextrose liquid medium. After 14 days of culture shaking (25 °C, 120 rpm), the mixture of fungal mycelia was homogenized by blender and then used as an inoculum.

### Experimental design

The pot experiment was performed using one inoculation status treatment (inoculated with *S. variegatus* or sterile inoculum) as a single-factor experiment under three drought intensity levels (non-drought, moderate drought, and severe drought). When the seedlings have finished acclimation period, all pots were randomly divided into 2 inoculation treatments: inoculated with *S. variegatus* and sterile inoculum, 30 pots per treatment. For ectomycorrhizal fungal inoculation, 30 mL inoculum was applied for the roots of *P. tabulaeformis*, whereas the non-mycorrhizal treatment received 30 mL autoclaved inoculum. Then all the pots were well watered and kept at 85–90% of the field capacity in the greenhouse at 25 °C with 12 h light per day for 4 weeks. In our previous field trials, we found that the field capacity was 20–40% under natural drought conditions on the Loess Plateau. After 4 weeks, each inoculation treatment was subjected to three levels (T1 non-drought: 80% of the field capacity, T2 moderate drought: 40% of the field capacity, and T3 severe drought: 20% of the field capacity) of drought intensity (Table 1), 10 pots per level. All pots were weighed and watered every day at 09:00 AM to maintain the water content at the desired levels. Trees were grown in the same greenhouse (average temperature was 25 °C and average relative humidity was 70%) and kept at stable water content for 60 days and then harvested.

### ECM fungal colonization

The ECM fungal colonization was measured on the basis of the method used in previous study [63], with minor modifications. Part of the fine roots were carefully washed with distilled water, bleached with 10% KOH at 90 °C for 24 h and then rinsed with distilled water, after which alkaline H_2_O_2_ was added to soften for 5 min. After the fine roots were washed with distilled water again, 1% HCl was added to acidify for 5 min. The washed fine roots were added with trypan blue dye (250 mL of lactic acid and glycerol, 0.5 g of trypan blue and 500 mL of water) and placed it in a water bath (90 °C) for 30 min. The roots were subsequently decolorized with lactic acid-glycerol (1:1) at 90 °C for 30 min. The colonization was counted according to the grid line intersection method under an optical microscope [63, 64].

### Leaf gas exchange parameters and water potential

Before the end of the experiment, seedlings with the same growth conditions were used to determine the gas exchange parameters including stomatal conductance (Gs), transpiration rate (T), and photosynthesis rate (A). The gas exchange parameters of the seedlings were measured using a LI-6400 Photosynthesis System (Licor Inc., Lincoln, NE, USA), and the photosynthesis system parameters were set as follows: the chamber area 2 cm^2^, the reference CO_2_ concentration was 385 ppm, the light environment was 1000 l mol m^− 2^ s^− 1^ using the 6400-2B red/blue LED light source. On sunny days from 9:00 to 11:30 AM, the youngest fully expanded leaves were selected for measurement. The water potential (Ψ) of *P. tabulaeformis* leaves was measured using the pressure chamber method [65].

### Morphology and biomass determination

After measuring the gas exchange parameters, the seedlings height and ground diameter of the *P. tabulaeformis* were measured, and the mortality was calculated. All the seedlings were divided into three parts, one part was used for root scanning, the other part was used for biomass determination, and another part is used for NSC content determination. One part of seedlings was divided into two parts (leaves and roots system) after harvested, placed them in a clear plastic bag and scanned it with a root scanner (STD1600 Epson, Long Beach, CA, USA). The root system index we measured were root length, root surface area, root average diameter, root volume, root forks and root tips. The scan results were analyzed by the software WinRHIZO™. The other part of seedlings was quickly cleansed after harvest, and the roots, stems, and leaves were collected separately for biomass determination. These tissues were dried at 75 °C for 48 h until constant weight.

### Non-structural carbohydrate determination

Another part of seedlings was divided into four parts: leaves, stems, coarse roots (> 2 mm), and fine roots (< 2 mm) after harvested. To limit the photosynthesis consumption on concentration of NSC in seedlings, the sampling time was completed within 2 h. After washing and thoroughly removing surface moisture, they were weighed and used to determine non-structural carbohydrate content. The collected samples were quickly heated under a microwave of 650 W for 90 s to prevent enzymatic carbohydrate reactions [66]. They were then dried to constant weight in an oven at 80 °C, and the dry weight was recorded. All dried tissues were ground until they passed smoothly through a 40-mesh sieve.

NSC concentration was defined as the sum of soluble sugar and starch content. According to the anthrone method [67, 68], some modifications were made to determine the NSC content. Precise 0.1000 g of samples from different organs was weighted and placed in 10 mL centrifuge tubes. Two milliliter 80% ethanol solution was added to the centrifuge tube, and then water bath at 80 °C for 30 min. After the solution was cooled to room temperature, the solution was centrifuged at 4800 rmin^− 1^ for 10 min. The supernatant was retained for determination of soluble sugar content. The precipitate was retained for determination of starch content [67]. The extraction was repeated 3 times. Added 2 mL of distilled water to the precipitate, gelatinized in a boiling water bath for 15 min. After the solution was cooled to room temperature, added 2 mL of 9.2 M HClO_4_ solution, shaken for 15 min, added 4 mL of distilled water, mixed and centrifuged at 4800 rmin^− 1^ for 10 min. After aspiration of the supernatant, there was a further extracted with 2 ml of 4.6 M HClO_4_. All supernatants were collected for determination of starch content. The absorbance of the solution after the sugar and starch reacted with the anthrone reagent was measured using a spectrophotometer at 625 nm. The content was calculated according to the standard curve and expressed as a % relative to the dry weight of the organ.

### Statistical analysis

All data were statistically analyzed using SPSS 25 (IBM® SPSS® Statistics) software. Repeated-measures analysis of variance (ANOVA) was used to determine the effects of drought intensity and ECM fungus (*S. variegatus*) on morphology, biomass, leaf gas exchange parameters, water potential, and NSC concentrations in each organ. Before ANOVA, levene’s test was used to test the homogeneity of the variance. The Tukey (HSD) test was used to determine significant differences (*P* < 0.05) in each indicator.

## Supplementary Information

**Additional file 1: Figure S1.** The phenotypes of *Pinus tabulaeformis* as influenced by ECM inoculation under different drought gradients. CK = No ECM fungi inoculation; ECMF = ECM fungi inoculation; T1 = non-drought stress; T2 = moderate drought stress; T3 = severe drought stress.

**Additional file 2: Figure S2.** The colonization of *Pinus tabulaeformis* by ECM at different levels of drought. CK = No ECM fungi inoculation; T1 = non-drought stress; T2 = moderate drought stress; T3 = severe drought stress.

## Data Availability

The datasets used and/or analysed during the current study are available from the corresponding author on reasonable request.

## Abbreviations

ECM: Ectomycorrhizal
NSC: Non-structural carbohydrate
*S. variegatus*: *Suillus Variegatus*
*P. tabulaeformis*: *Pinus tabulaeformis*
PDA: Potato dextrose agar
CK: No ECM fungi inoculation
ECMF: ECM fungi inoculation
T1: Non-drought stress
T2: Moderate drought stress
T3: Severe drought stress
A: Photosynthesis rate
Gs: Stomatal conductance
T: Transpiration rate
