# Supplementary figures and images for: Effects of ectomycorrhizal fungi (Suillus variegatus) on the growth, hydraulic function, and non-structural carbohydrates of Pinus tabulaeformis under drought stress
Source: BMC Plant Biol. 2021 Apr 10;21:171. doi: 10.1186/s12870-021-02945-3 (PMC8035767; doi:10.1186/s12870-021-02945-3)

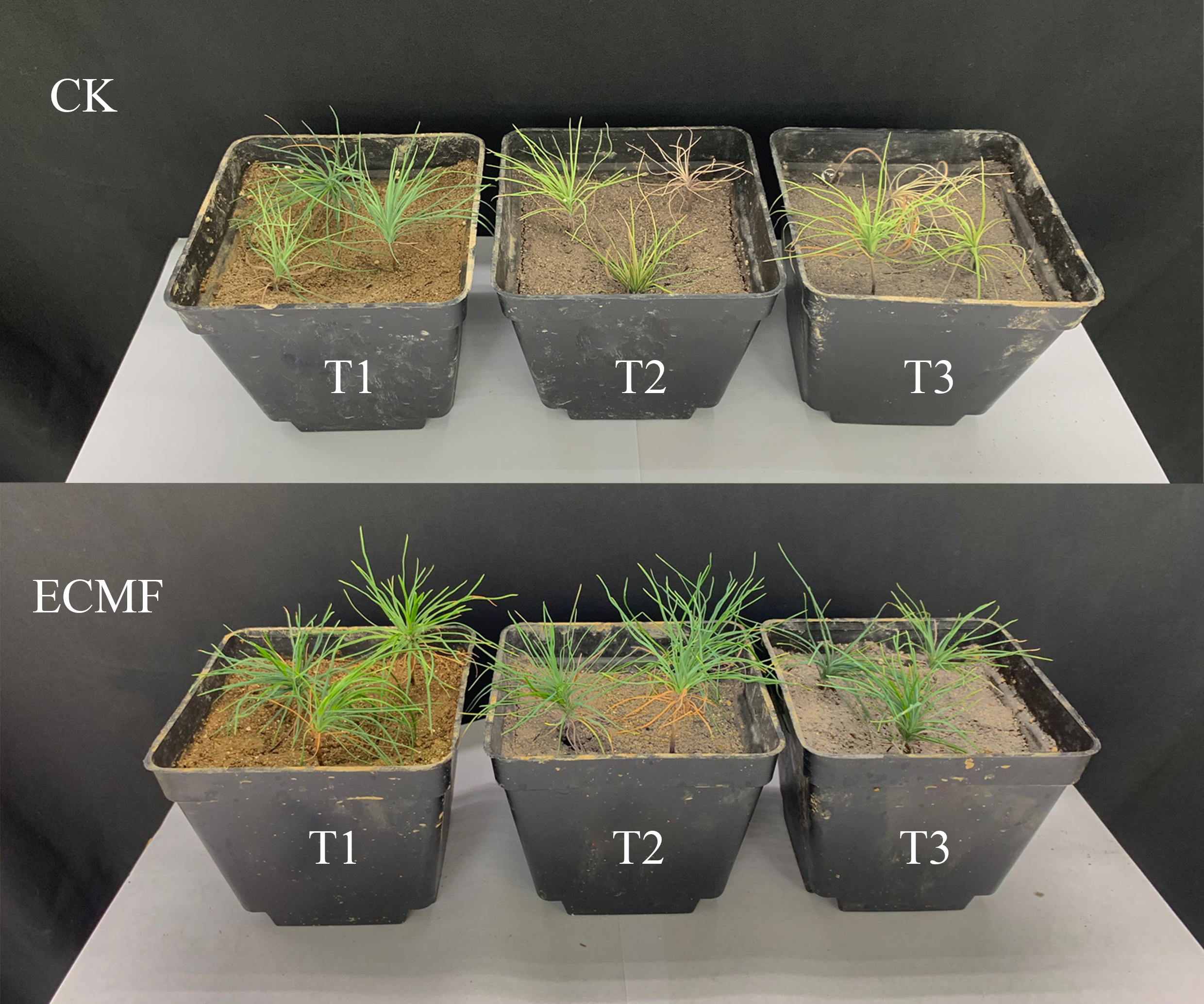

Supplement: Supplementary file 1 — Additional file 1: Figure S1. The phenotypes of Pinus tabulaeformis as influenced by ECM inoculation under different drought gradients. CK = No ECM fungi inoculation; ECMF = ECM fungi inoculation; T1 = non-drought stress; T2 = moderate drought stress; T3 = severe drought stress. [file 12870_2021_2945_MOESM1_ESM.tif]

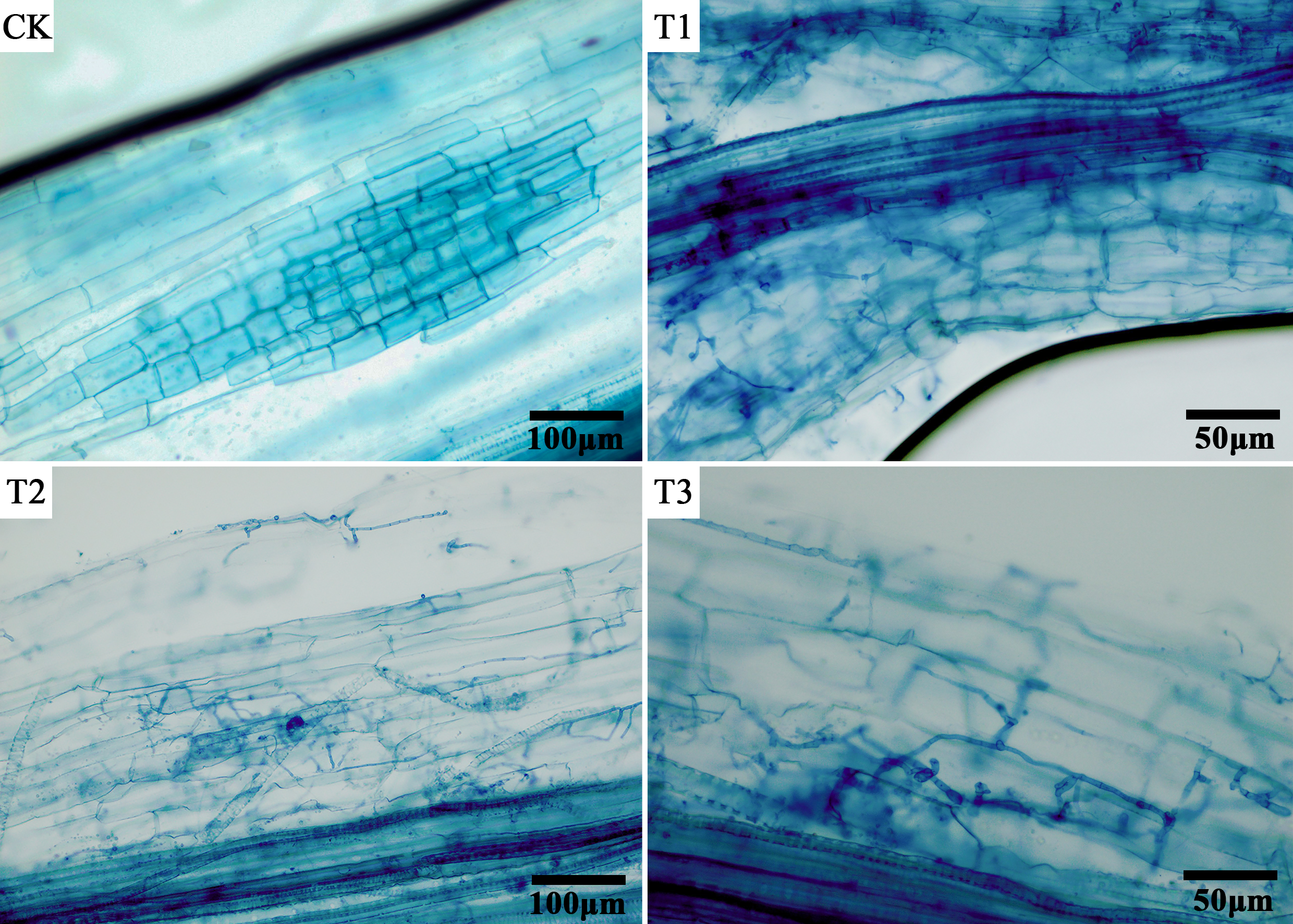

Supplement: Supplementary file 2 — Additional file 2: Figure S2. The colonization of Pinus tabulaeformis by ECM at different levels of drought. CK = No ECM fungi inoculation; T1 = non-drought stress; T2 = moderate drought stress; T3 = severe drought stress. [file 12870_2021_2945_MOESM2_ESM.tif]
